# Frequency and Risk Factors of Surgical Site Infection among Sudanese Patients with Oral Squamous Cell Carcinoma

**DOI:** 10.1155/2024/7525831

**Published:** 2024-02-08

**Authors:** Mohamed Elfayeg, Ahmed Suleiman, Yousif Eltohami

**Affiliations:** ^1^Khartoum Teaching Dental Hospital, Khartoum, Sudan; ^2^Faculty of Dentistry, University of Khartoum, Khartoum, Sudan

## Abstract

**Background:**

In Sudan, patients with oral squamous cell carcinoma (OSCC) presented lately in advanced stages. Surgical site infection (SSI) is one of the most common complications of surgical treatment of OSCC which significantly affects the clinical outcomes. The present study aimed to assess the frequency and risk factors of postoperative surgical site infection among OSCC patients underwent surgery at Khartoum Teaching Dental Hospital (KTDH).

**Methods:**

This is a prospective, analytical, hospital-based study conducted at KTDH during the period from 2022 to 2023. Patients with OSCC were surgically treated and assessed carefully for the development of the SSI.

**Results:**

Sixty patients were enrolled in the present study. Twenty-nine (48.3%) patients were above 61 years, with the predominance of males with 42 (70%) patients. The most involved site of OSCC was the lower gingivolabial region in 35 (39.3%) patients. Forty-seven (78%) patients were in advanced stages III and IV. Forty-five (80%) patients had modified radical neck dissection. Blood transfusion was administered in 50 (83.3%) patients. Twenty-six (43.4%) patients developed SSI; 15 (57.7%) patients of them were Toombak dippers. Development of SSI was found to be significantly associated with the tumour site (*P* value 0.9), clinical stage (*P* value 0.6), the number of transfused blood units (*P* value 0.04), and the duration of hospital stay (*P* value 0.04). In contrast, use of sutures for wound closure was associated with a reduced risk of developing SSI (*P* value 0.005).

**Conclusion:**

Surgical site infection was found in 43.4% of the OSCC patients. It was associated with advanced clinical stage and tumour site. Minimizing the number of blood units transfused intraoperatively, we decrease the duration of hospital stay and the use of sutures for wound closure decreases the risk of SSI significantly.

## 1. Introduction

Damage to the skin or mucous membranes permitting bacterial entry into typically sterile areas can lead to various infections, including invasive surgical site infections (SSIs) [1]. Surgical site infection has replaced the previously used term surgical wound infection. The name SSI was introduced by the US Centre for Disease Control and Prevention (CDC) in 1992. According to the CDC definition, SSIs can be divided as follows: 1. Superficial: developing within 30 days since surgery and involving skin and subcutaneous tissue; 2. Deep: developing after 30 days or within one year if a foreign body was implanted and involving fascia and muscles; 3. Organ or body cavity infection near the surgical site: developing within 30 days or one year if a foreign body was implanted [2]. SSI stands out as the most extensively researched healthcare-associated infection (HAI) and is the predominant HAI in developing regions. Globally, SSIs correlate with heightened levels of illness and death, resulting in consequences such as additional surgeries, reduced quality of life, prolonged antibiotic usage and rehabilitation, and subsequent losses in work and productivity [1]. The occurrence of a surgical site infection (SSI) significantly amplifies both the clinical and economic challenges associated with surgery. SSIs have adverse effects on both the physical and mental well-being of patients. Indirect costs associated with infection include heightened patient morbidity, increased mortality rates, and a loss of earnings during the recovery period. Patients may also face intangible costs, such as pain and anxiety. Moreover, delayed wound healing and an elevated susceptibility to secondary complications, such as bacteraemia, can further impact patients. Prolonged hospitalization and the associated absence from home and work may cause distress to both the patient and their family members. As a result, the development of an SSI has been demonstrated to negatively impact the health-related quality of life (HRQoL) of patients, manifesting in increased morbidity and prolonged hospitalization. In addition, some patients may necessitate reoperation following the development of an SSI, leading to substantial additional financial burden [3]. The large defect formed by resection of advanced oral cancer results in severe dysfunction of swallowing and breathing as well as cosmetic disorders. Recent advances in microvascular free flap surgery made it possible to reconstruct the huge defect according to its complicated morphology. However, in oral cancer surgery, the duration of surgery and the amount of blood loss are increased, and there is a greater degree of surgical invasion, which may induce serious postoperative complications including SSIs [4].

In the field of oral surgery, patients diagnosed with oral squamous cell carcinoma (OSCC) frequently experience surgical site infections (SSIs) due to the prominence of surgery as the primary therapeutic intervention, particularly in advanced cases. There has been an increasing focus in recent years on assessing the likelihood of postoperative SSIs by employing various risk-stratification criteria or systems. A diverse array of risk factors has been documented across various surgical specialties, including plastic, cardiothoracic, gastrointestinal, and urinary surgeries [5]. Among these, old age has always been considered one of the most prominent risk factors for the development of SSIs. Surgical site infections are currently believed to account for 11% of all nosocomial infections in patients older than 65 years [6].

The present study aimed to assess the frequency and risk factors of postoperative surgical site infection in Sudanese OSCC patients operated at Khartoum Teaching Dental Hospital.

## 2. Materials and Methods

### 2.1. Study Design and Data Collection

This is a prospective, analytical, hospital-based study that has been performed at Khartoum Teaching Dental Hospital in Khartoum state. The study was conducted from January 2022 to January 2023. Any Sudanese patient diagnosed with oral squamous cell carcinoma who underwent surgery during the study period was included in this study. In contrast, patients with oral cancer other than oral squamous cell carcinoma, those with squamous cell carcinoma receiving neoadjuvant treatment, patients who presented with recent or second primary tumours, and patients whose records are incomplete were excluded from the study. All the included patients in the study underwent surgical excision of the tumour, neck dissection, and flap reconstruction, and all the procedures were performed simultaneously in the study population.

The data were collected by collecting data from patients' records upon consent, and information retrieved included sociodemographic data, clinical presentation, anatomical site, tumor stage, histopathological type and grade, and presence of metastasis (nodal, distant). A baseline-administered questionnaire containing study variables was used by the principal investigator.

A thorough clinical and radiographical examination was done along with histopathological analysis, the microbiological analysis was conducted in Khartoum teaching dental hospital, and the histopathological analysis was conducted in the Department of Histopathology and Cytology at Khartoum University.

The clinical stage of the disease was assigned to each patient by using TNM (AJCC cancer staging manual); this is a staging system which is an expression of the anatomical extent of the disease based on the extent of the primary tumor (T), absence or presence of and extent of regional lymph node metastasis (N), and absence or presence of distant metastasis. Depending on the site, biopsy specimens were obtained by excisional or incisional biopsies. Tissue specimens were submitted for histopathological examination. Frozen section study was not available. All the patients were diagnosed with OSCC, and since nearly all individuals diagnosed with oral squamous cell carcinoma and using Sudanese snuff “Toombak” were Sudanese, the study included solely Sudanese patients.

The operating theater underwent comprehensive cleaning and disinfection procedures before each surgery, between patient transitions, and postsurgery. This process entails initial cleansing with warm soapy water, followed by wiping using a cloth drenched in clean water. Ultimately, a 1% solution of sodium hypochlorite serves as the final disinfectant agent. All the patients underwent wide surgical resection with safety margins, neck dissection surgery, and reconstruction surgery. Following the surgery, the excised surgical specimen was sent to the Histopathology and Cytology department at Khartoum University to be examined by an oral pathologist. Patients were admitted 24 hours prior to surgery and postoperatively for a period ranging from (3 days- >16 days). Patients were monitored daily; all the clinical data were collected for analysis.

### 2.2. Statistical Analysis

The data were analysed using SPSS statistical package software version 28.0, Microsoft Excel sheets to group the data. Pie charts, bar graphs, and custom table have been used to visualize data. The statistical tests have been used in descriptive analysis for categorical variables which were mean, frequencies, chi square, and *T*-test for numerical values. Stepwise regression was performed to predict the risk factors.

### 2.3. Ethical Considerations

Approvals were obtained from the Sudan Medical Specialization Board, Research Committee Board, EDC Ethical Committee Board, the Ministry of Health, and Khartoum Teaching Dental Hospital administration. Confidentiality and privacy of the data have been ensured by using serial numbers, and written consent from the patients was obtained.

## 3. Results

In this hospital-based study involving 60 patients diagnosed with oral squamous cell carcinoma, the gender distribution showed that 18 (30%) were females, while 42 (70%) were males. Most patients were over 61 years old, with 33 (55.6%) being Toombak dippers, and 19 (57.58%) among them had been dipping for over 30 years. The study encompassed various age groups ranging from 20 years old to above 60, with the majority being over 60, and only three (5.0%) were younger than 30.

**Figure 1:**
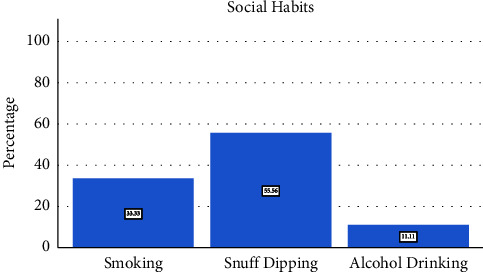
The social habits of the patients.

**Figure 2:**
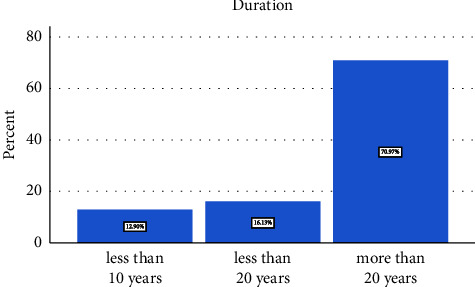
Duration of snuff dipping.

Out of the 60 patients, 26 (43.4%) developed surgical site infections (SSI), with the majority of those affected being over 61 years old (15 patients - 57.7%). Social habits analysis revealed that 33 (55.56%) were snuff dippers, 20 (33.33%) were smokers, and only seven (11.11%) were alcohol drinkers (Figure 1). Among those who developed SSI, 17 (65.4%) had social habits. The duration of snuff dipping among the participants showed that 23 (70.97%) had been doing so for more than 20 years (Figure 2).

Medical history indicated that eight (38.1%) participants had diabetes mellitus, eight (38.1%) had cardiovascular disease, two (9.5%) had HBV/HCV, two (9.5%) had liver disease, and one was pregnant. Squamous cell carcinoma primarily affected the mandibular gingivolabial area in 35 (39.3%) patients. Most patients (47–78%) were in advanced stage III or IV.

Regarding surgical procedures, sutures were used in 40 (66.7%) patients for skin closure, while staples alone or in combination with sutures were used in the remaining cases. Blood transfusion was administered to 50 (83.3%) patients during surgery, with varying pint quantities.

Postoperatively, 26 (43.4%) patients developed SSI, and the duration of hospital admission varied, with 37 (61.6%) patients staying for more than 16 days (Table 1). The onset of SSI mostly occurred during the third week postoperatively (21 patients: 35%) (Table 2).

Association analysis showed a higher incidence of infection among patients with certain medical conditions, primarily in the mandible (21 patients - 80.8%). Tumor staging indicated that 24 (92.3%) of those who developed SSI were in stages III or IV.

Statistical analysis revealed that sutures for wound closure were associated with a reduced risk of infection, while the number of blood transfusion units and longer hospital stays increased the risk of developing SSI (Table 3).

## 4. Discussion

Postoperative infections after oral cavity cancer surgery occur at a concerning rate, nearing approximately 40%. As highlighted by Mascarella et al. in their study, oral cancer patients who developed postoperative infections were 2.5 times more prone to mortality compared to those without infections, after adjusting for different influencing factors. This trend of worse long-term survival associated with postoperative infections has also been observed in patients undergoing lung cancer surgery, as reported in population-based studies. Similarly, individuals with head and neck cancer undergoing ablative surgery faced an elevated risk of recurrence following postoperative wound infections [7].

Surgical site infection (SSI) is a known postoperative complication and is defined as “the development of an infection in a surgical wound within 30 days of the procedure.” It is frequently observed in oral surgery, typically manifesting as a straightforward infection marked by pain, pus drainage, wound opening, and general symptoms. If left untreated, it may progress to systemic infection and, in severe cases, even result in septicaemia and mortality [8].

In this study, the incidence of SSI was examined and 21 potential risk factors were scrutinized through both univariate and multivariate analyses to establish their connection with SSI. These factors were categorized as preoperative, intraoperative, and postoperative to streamline analysis. Out of these factors, only five were deemed statistically significant: tumor location, disease stage, wound closure materials, intraoperative blood transfusion, and the length of hospital stay following the operation. Concerning the occurrence of SSI and the primary factors associated with it, this study encompassed 60 patients, all of whom underwent major surgery for OSCC. The observed SSI rate was 43.3%, close to the findings of Lin et al., who conducted a multivariate analysis involving 173 comparable surgical cases, in which the rate of SSI development was 38.7% [9]. Notably, this rate was notably higher than the 10.9% reported by Cunha et al. [10] and the 15.9% reported by Shi et al. [8].

Commencing with preoperative risk factors, demographic data were gathered from all patients, with particular emphasis on age and gender. Advanced age has long been recognized as a prominent risk factor. In this study, among the 26 patients who experienced SSI, 57.7% were aged over 60. However, in the multivariate analysis, no substantial correlation was established. Presently, surgical site infections are estimated to contribute to 11% of all hospital-acquired infections in individuals over 65 years old [6].

Regarding risk factors, social habits rank prominently in the literature, encompassing smoking, snuff dipping, and alcohol intake. These habits are widely recognized as significant contributors to OSCC development and are also implicated in various mechanisms leading to SSI. Toombak, a Sudanese smokeless tobacco used orally, contains nicotine and other compounds linked to severe health consequences, thereby heightening the risk of surgical site infection [11].

Concerning the preoperative coexisting conditions among the patients in this study, 35% presented with medical comorbidities, with diabetes mellitus (D.M) and cardiovascular disease (CVD) being the most prevalent. Diabetes is known to induce microvasculopathy and immunosuppression, hindering wound healing and escalating the SSI risk [12]. CDC guidelines advocate for perioperative glycaemic control, aiming for a blood glucose target level below 200 mg/dl, applicable to patients with or without diabetes mellitus, to prevent SSI. Lee et al. reported a significant association between diabetes (*P*=0.002) and an increased incidence of SSI [13]. Similarly, Shi et al.'s analysis showed a positive link between diabetes mellitus (*P*=0.008) and SSI [14]. Conversely, Ogihara et al. documented that diabetes did not show a significant association with SSI [15]. While Lotfi et al. identified medical comorbidities as a notable risk factor for SSI development [16], Belusic-Gobic et al. found no statistical significance between medical comorbidities and SSI development [17].

Another contributing factor influencing the postoperative SSI rate is the location and size of the tumor in the head and neck, as well as its spread into neighbouring tissues. In this study, the most affected site of OSCC among the patient group was the lower gingivolabial area in 35 (39.3%) patients, followed by 12 (20%) patients in the lips, and seven (11.6%) patients in the upper gingivolabial area. Nearly all patients who developed SSI had the upper and/or lower gingivolabial area as the primary site of the tumor. In contrast to these findings, Hakan Coskun et al. concluded that no statistically significant difference was evident based on tumor localization (*P* > 0.05) [18]. However, Pecorari et al. highlighted the tumor site as a potential risk factor, noting slight differences in specific anatomical sites, and recommended further large-scale studies to ensure reliable results from multivariate analyses, aligning more with the outcomes observed in this study [19].

Concerning the clinical tumor stage as per the current AJCC/UICC TNM staging (8th edition, 2017) for OSCC [13], most patients in this study, 47 (78%), were in advanced stages III or IV, while a smaller proportion, 13 (21.7%), were in the earlier stages I and II. Notably, most patients who developed SSI were in the advanced stages III and IV (*P* value 0.9). Elevated tumor stage notably heightens the risk of postoperative surgical site infection. Typically, neoplasms impact the immune system, inducing immunosuppression by affecting lymphocyte function. This effect renders the body more vulnerable to infections. Furthermore, extensive surgery is often required to excise larger tumors, particularly in neck dissection procedures, resulting in additional wounds [15, 20, 21].

In this study, most patients were diagnosed at an advanced stage of cancer, aligning with findings from other research in developing nations. Late presentations in these regions could stem from factors such as lack of awareness, poverty, limited healthcare access, and patients seeking advice from traditional healers or using traditional remedies. Identifying primary cancer at an early stage significantly enhances the likelihood of successful treatment and, consequently, better chances of survival [22].

Coskun et al. noted, similar to this study, that wound infection rates are notably higher in advanced tumors. Their observation indicated that stage IV tumors had a heightened postoperative wound infection rate compared to early stages (*P*=0.002) [18]. Pecorari et al. reported infection rates of 10.9% and 36.0% in patients with early (stage I-II) and advanced (stage III-IV) cancer, respectively (*P*=0.048) [19]. Lotfi et al. documented that most patients experiencing SSI were at clinical stages III and IV (69.4%). They diagnosed surgical site infections in 100 (38.8%) patients. Their analysis of the T stage variable revealed that patients with T3 or T4 tumors exhibited higher SSI rates (44.7%) compared to patients with tumors at stages T1 and T2 (28.9%, P 0.018), aligning with the findings of this current study [16].

Reconstructive surgery following oral cancer resection is gradually becoming a customary procedure due to the resultant defect. However, reconstruction significantly influences the postoperative infection rate due to the heightened complexity of the operation, extended duration, larger tissue defects, the addition of a secondary wound (donor site), and increased blood loss, all contributing to the heightened risk of SSI. Microvascular free flap reconstruction, while more functional for addressing intricate defects compared to regional pedicle flaps, comes with a set of complications involving both donor and recipient sites, notably SSI with an incidence rate of 62.12%. Complication rates tend to rise when bone is transferred as part of the flap. Moreover, it has been theorized that SSI may lead to increased cancer recurrence rates through immunological mechanisms [8, 9, 23]. Furthermore, complex reconstructions, closure of suture lines under tension, or failure to achieve a watertight oral seal heighten the likelihood of mucocutaneous fistula and wound infection [18].

Shi et al. investigated the risk factors for surgical site infection after major oral oncological surgery, revealing a significant association between SSI and flap reconstruction, as well as the specific type of flap employed (*P* < 0.001) [8]. Meanwhile, Cloke et al. determined that wound infection is a frequent and challenging issue but did not identify any statistically significant associations between the variables studied, including surgical reconstruction, and SSI [23].

Following tumor removal, an evaluation was conducted on the materials used for wound closure, specifically comparing the use of sutures versus surgical staples or a combination of both. In this study, sutures were employed in 66.7% of patients, while a combination of staples and sutures was utilized in 31.7% of patients, and only one instance used staples alone. Notably, the use of sutures was linked to a reduced risk of infection (*P* value 0.005, odds ratio 0.06). Van de Kuit et al. found no significant difference in SSI risk between patients treated with staples (*n* = 557) versus sutures (*n* = 573) (RR: 1.70, 95% CI: 0.94–3.08, *I*^2^ = 16%). However, their analysis with a low bias risk revealed a notably higher SSI risk in patients with staples (*n* = 331) compared to those with sutures, aligning with the findings of this present study [24].

The ongoing discussion surrounding the use of sutures versus staples in surgical wound closure persists despite the documented lower infection rates associated with sutures in the literature. This debate might stem from differences in closure techniques, where staples are externally applied while sutures penetrate the skin and are tied externally, as well as disparities in removal timelines between sutures and staples [25].

In this study, blood transfusion during surgery and the quantity transfused were also linked to a 1.9-fold increased risk of developing infection (*P* value 0.04), establishing it as a significant risk factor. Notably, blood transfusion potentially impacts the recipient's immune system by theoretically suppressing its immune response, referred to as transfusion-associated immunomodulation (TRIM). Several studies have endeavored to explore this phenomenon, yet conclusive results have not surfaced [26]. Among patients who experienced SSI, only one did not receive blood during surgery. Of those who did, 53.8% received 2-3 pints, while 46.2% received 2-3 pints. Cheng et al. noted an increase in SSI incidence with higher volumes of blood transfusion (measured in the number of pints). They found that the occurrence of SSI among patients who received transfusions (10.7%) was notably higher compared to those who did not (3.0%), suggesting a higher susceptibility to SSI among surgical patients who undergo blood transfusions, which aligns with the findings in this present study [14]. Conversely, Margita Belusic-Gobic et al. did not detect any statistically significant link between blood transfusion and wound infection, including the quantity of blood units received during surgery [17]. Similarly, Lin et al. concluded that blood transfusion did not significantly contribute to the incidence of SSIs [9].

The extended duration of a surgical procedure as a risk factor for SSI is extensively documented in the literature. This is mainly linked to the increased exposure to microorganisms in the surgical environment [14]. The length of surgery is influenced by the extent of the disease, the intricacy of the procedure, and the necessity for reconstruction. In this current study, 84.6% of patients who developed SSI underwent surgery lasting 3-4 hours, while 15.4% were in surgery for 5-6 hours [27].

In the multivariate logistic regression analysis conducted by Ma et al., operation time (*P* = 0.0003) emerged as significantly and independently associated with the development of SSI [12]. Likewise, Cheng et al. identified an operative duration exceeding 120 minutes (*P* = 0.001) as an independent factor linked to the occurrence of SSI [28]. Similarly, Ansari et al. observed a higher incidence of SSI associated with longer surgical durations [29]. Conversely, Penel et al. reported no correlation between the risk of SSI and the duration of the procedure [30], while Cloke et al. found no statistically significant association between the duration of surgery and SSI [23].

Research has highlighted the reciprocal relationship between SSI and the duration of postoperative hospital stays, where SSI can prolong hospitalization and is also influenced by it. The hospital environment, known for harbouring infections (nosocomial infections), underscores the importance of early discharge when suitable to reduce postoperative SSI rates. The postoperative hospital stay was categorized into four groups: 3–5 days, 6–10 days, 11–15 days, and more than 16 days. In this study, 88.5% of patients who developed an infection were hospitalized for over 16 days. Prolonged hospital stays were associated with a 1.9-fold increased risk of developing SSI (*P* value 0.04), particularly for durations surpassing 16 days. Comparable findings were reported by Pecorari et al., who observed SSI in 22.6% of cases, noting that patients experiencing SSI had significantly longer median hospital stays (38 vs. 9 days) [19]. Edin Mujagic et al. explored the relationship between hospital length of stay (LOS) and SSI among 4596 patients, with 234 (5.1%) experiencing SSI. Their findings indicated no significant independent association between preoperative LOS and SSI risk, while SSI and postoperative LOS were notably linked [31].

Numerous studies have been undertaken concerning the prevention of SSI, commencing with the cleaning and disinfection of the operating room, and Bosco et al. noted in their study that the implementation of UVC-D has enhanced environmental disinfection, effectively reducing the risk of crosscontamination in operating theatres [32].

The adoption of evidence-based collective preventive measures, known as the “care bundle” approach, represents a factor worth considering. Fernandez-Prada et al. documented the use of bundles in patients undergoing vascular surgery, incorporating various components: (1) removal of body hair using clippers, (2) preoperative cleansing with chlorhexidine soap, (3) preparation of the surgical area using alcoholic chlorhexidine 2%, (4) ensuring adequacy of antimicrobial prophylaxis, (5) managing intraoperative glycaemic and central temperature control, and (6) managing postoperative glycaemic and central temperature control [33].

Another aspect highlighted involves hand washing and scrubbing before surgery. Stillo et al. highlighted that washing with ethanol, hydrogen peroxide, and glycerol (EPG) showed superior efficacy in reducing colony-forming units (CFU). Nonetheless, they emphasized that antiseptic hand washing alone should not be the sole measure to mitigate infections. Their research underscored the significance of the drying phase in presurgical preparation. Consequently, effective hand hygiene should be integrated into a comprehensive strategy aimed at monitoring and managing healthcare-associated infections [34].

While the present study did not reveal any statistical association between the site/onset of infection and the occurrence of SSI, it is noteworthy that most patients who developed an infection were affected at the primary tumor site. In addition, among the infected patients, 21 (35%) experienced SSI during the 3rd week postoperatively, suggesting a potential correlation, albeit not strongly suggestive. In Cloke et al.'s study, they observed 17 infections in the neck, two in the cheek, one in the mouth, and one concurrently in the mouth and neck [23].

The study's limitations encompassed the retrospective nature of the analysed data, leading to a less than optimal examination of the risk factors associated with SSI development in patients. In addition, the study suffered from a small sample size. Future investigations with larger sample sizes could potentially strengthen the results observed in this study, adding further support to the outcomes obtained.

## 5. Conclusion

The current study found that surgical site infections affected 43.4% of patients. Multivariate analysis revealed that a higher number of blood transfusion units and an extended hospital stay correlated with an increased risk of SSI development. Conversely, using sutures to close wounds was linked to a decreased risk of SSI occurrence. [14].

## Figures and Tables

**Table 1:** Duration of hospital stay.

Duration of hospital stay		
	*N*	%

3–5 days	1	1.7
6–10 days	1	1.7
11–15 days	21	35.0
>16 days	37	61.6

**Table 2:** Onset of surgical site infection.

Onset of the infection		
	*N*	%

1st week postoperative	1	1.7
2nd week postoperative	4	6.7
3rd week postoperative	21	35
No infection developed	0	56.6

**Table 3:** Multivariate logistic regression.

Variable	Significance (*P* value)	Odds ratio
Site	0.9	—
Clinical stage	0.6	1.5
Material used		0.02
Staple	1	—
Sutures	**0.005**	**0.06**
Both	Reference	
Blood transfusion	0.4	2
Number of units	**0.04**	**1.9**
Duration of hospital stay	**0.04**	**1.9**

Bold values indicates the *P* value significant statistically as less than 0.05.

## Data Availability

The data used to support the findings of this study are available from the corresponding author upon request.
